# Laminin α2, α4, and α5 Chains Positively Regulate Migration and Survival of Oligodendrocyte Precursor Cells

**DOI:** 10.1038/s41598-019-56488-7

**Published:** 2019-12-27

**Authors:** Nobuharu Suzuki, Mai Hyodo, Chikako Hayashi, Yo Mabuchi, Kaori Sekimoto, Chinami Onchi, Kiyotoshi Sekiguchi, Chihiro Akazawa

**Affiliations:** 1 Department of Molecular and Cellular Biology, Graduate School of Medical and Dental Sciences, Tokyo Medical and Dental University (TMDU), Tokyo, Japan; 2 Department of Biochemistry and Biophysics, Graduate School of Health Care Sciences, TMDU, Tokyo, Japan; 3 Department of Biochemistry and Biophysics, Graduate School of Medical and Dental Sciences, TMDU, Tokyo, Japan; 4 Division of Matrixome Research and Application, Institute for Protein Research, Osaka University, Suita, Osaka Japan

**Keywords:** Glial progenitors, Oligodendrocyte

## Abstract

In the developing central nervous system (CNS), oligodendrocyte precursor cells (OPCs) migrate along blood vessels and are widely distributed in the CNS. Meanwhile, OPCs require survival factors from the extracellular microenvironment. In other tissues, laminins, heterotrimetric (αβγ) extracellular matrix proteins, promote cell migration and survival. However, the expression pattern and functions of laminins in OPC development remain poorly understood. In the present study, we first investigated the expression of laminin α chains, which bind to cell surface receptors such as integrins, in the postnatal murine brain. We found that laminin α1, α2, α4, and α5 chains were expressed around blood vessels and OPCs attached the laminin α chain-positive vessels. We then evaluated the effect of these laminins on OPCs activity using recombinant laminin E8s (LME8s) that are minimally active fragments of the laminin isoforms. OPCs attached on LM211E8, LM411E8, and LM511E8, containing laminin α2, α4, and α5 chains, respectively, through integrin β1. Further, these three LME8s promoted migration of OPCs, and OPC survival was prolonged on either LM411E8 or LM511E8 via the activation of focal adhesion kinase. Together, our findings suggest that laminins expressed surrounding blood vessels positively regulate migration and survival of OPCs through the integrin β1-FAK pathway.

## Introduction

Oligodendrocyte precursor cells (OPCs), alternatively called O2A cells, NG2 cells, or polydendrocytes, are one of glia cells in the central nervous system (CNS) and are differentiated into oligodendrocytes in the white matter for myelination, which accomplishes rapid salutatory conduction of nerve impulses and contribute to axonal integrity^1^. OPCs can differentiate into not only oligodendrocytes but also astrocytes in the ventral cortex^2^, indicating that OPCs are required for providing these glia cells depending on conditions of tissues. In particular, under the pathological condition with demyelination, the replenishment of OPCs is required for remyelination. OPCs arise from the ventricular/subventricular zone (VZ/SVZ) in the CNS tissues and migrate to the targeted areas for their differentiation^3^. Throughout this dynamic process, OPCs need to obtain survival signal from growth factors or cytokines and from cell adhesion on extracellular matrix proteins.

Laminins are extracellular matrix proteins in basement membranes and regulate cell proliferation, migration, differentiation/morphogenesis, and survival in various tissues^4,5^. Laminins are one of the major basement membrane proteins and composed of three different subunits, α, β, and γ chains (e.g.: laminin, consisting of α1, β1, and γ1 chains, is called laminin-111). To date, five α, four β, and three γ chains have been identified, and at least 19 laminin isoforms are found *in vivo* with various combinations of the trimetric chain assembly^4,5^. Laminins bind to specific cellular receptors, including integrins, through the C-terminal globular domain of the α chains, and exert various biological activities via cell adhesion^4^. A previous study revealed that the expression of laminin β1 and γ1 chains, which are components of major laminin isoforms, was analyzed in the brain tissue at the early postnatal stage, when oligodendrogenesis dynamically occurs, and OPC survival was reduced in laminin α2 deficient mice^6^. However, the expression pattern of laminin α chains in the postnatal brain and the function of the other α chains in OPC development remain unknown.

In this study, we investigated the expression pattern and biological activities of laminin α chains in OPCs using anti-laminin α chain specific antibodies and recombinant laminin E8 fragments (LME8s) that possess the integrin binding activity equal to intact laminins, respectively^7^. Also, we used *Sox10*-Venus mice that express a fluorescent protein Venus specifically in oligodendrocyte lineage cells in the CNS^8,9^. We found that laminin α1, α2, α4, and α5 chains were expressed around blood vessels in the brain tissue at the postnatal stage. Further, LME8s containing laminin α2, α4, and α5 chains promoted OPC attachment and migration, and OPC survival was activated on α4 and α5 chains via focal adhesion kinase (FAK). To our knowledge, this is the first study to evaluate all of the laminin α chains for OPC development and to reveal their activities.

## Results

### Expression of laminin α chains in the murine brain at the early postnatal stage

**Figure 1:**
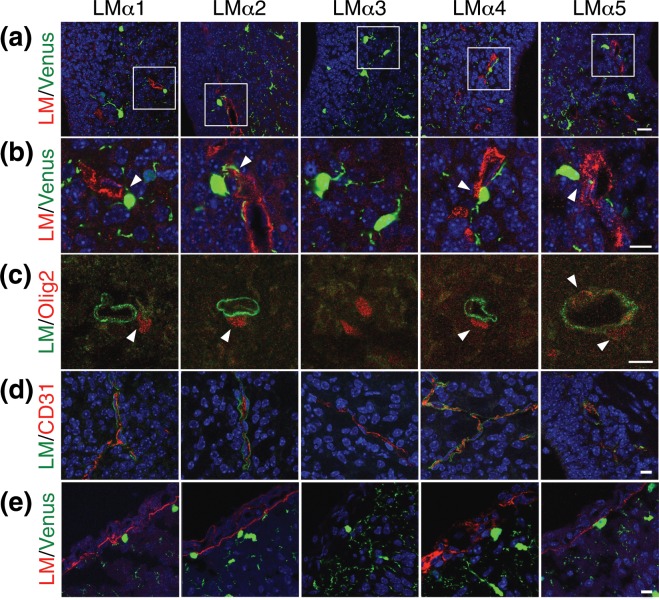
Expression of laminin α chains in P2 *Sox10*-Venus and WT mouse brains. (**a**) Immunohistochemistry of laminin α chains (red) in the ventricular/subventricular zone of P2 *Sox10*-Venus mouse brains. (**b**) Higher magnification images of the boxes in (**a**). (**c**) Immunohistochemistry of laminin α chains (green) and Olig2 (red) in P2 WT mouse brains. (**d**) Immunohistochemistry of laminin α chains (green) and CD31 (red) in P2 WT mouse brains. (**e**) Immunohistochemistry of laminin α chains (red) in the pia of P2 *Sox10*-Venus mouse brains. DAPI staining (blue) was used to visualize nuclei in (**a,b,d,e**). Arrowheads: attachment of OPC to vessel. Scale bars: 20 μm (**a**); 10 μm (**b–e**). LM: laminin.

We first assessed which laminin α chains were associated with OPCs in the postnatal mouse brain. For analyzing the expression of five laminin α chains in the brain, we used postnatal day (P) 2 *Sox10*-Venus mice, whose Venus positive cells are OPCs^9^, and P2 WT mice. Using the tissue sections, immunostaining with antibodies against laminin α1, α2, α3, α4, and α5 chains was performed. As a result, the expression of laminin α1, α2, α4, and α5 chains, but not α3 chain, was observed in basement membranes surrounding blood vessels, and Venus positive OPCs contacted the vessels that expressed these laminin α chains (Fig. 1a,b). We confirmed these results by immunostaining with antibodies against laminin α chains and an OPC marker Olig2 (Fig. 1c) and identified blood vessels using an antibody against an endothelial cell marker CD31 in the WT tissues (Fig. 1d). We also observed that laminin α4 chain was expressed around all of the CD31-positive vessels, while the percentages of laminin α1-, α2-, and α5-positive vessels out of all the vessels were 4.94%, 15.09%, and 15.75%, respectively. In addition, the expression of laminin α1, α2, α4, and α5 chains was found in pia and neighbor vessels, and some of Venus positive OPCs attached to the laminin-positive pia (Fig. 1e). These results suggest that laminin α1, α2, α4, and α5 chains interact with OPCs and may have functions in OPC biological activities.

### OPC attachment activity of LME8s

**Figure 2:**
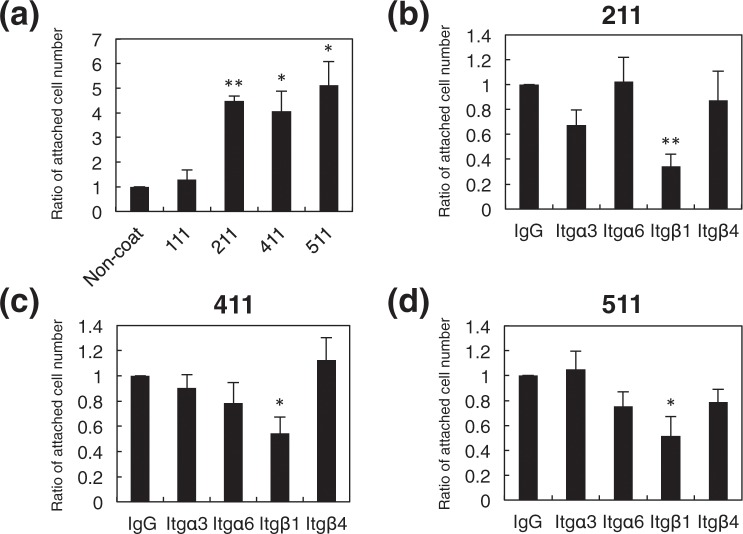
Cell attachment activity of OPCs on laminin E8 fragments. (**a**) Ratio of attached cell numbers on recombinant laminin E8 fragments (LM111E8, LM211E8, LM411E8, and LM511E8). The number of attached cells on non-coat as a control was set as 1.0. Error bars, s.e.m. (*p < 0.05; **p < 0.01, *t* test). (**b–d**) Ratio of attached cell numbers on LM211E8 (**b**), LM411E8 (**c**), and LM511 (**d**) with each inhibitory antibody. The number of attached cells with normal IgG as a control was set as 1.0. Error bars, s.e.m. (*p < 0.05; **p < 0.01, *t* test). At least triplicate experiments were independently performed. 111: LM111E8; 211: LM211E8; 411: LM411E8; 511: LM511E8; Itg: integrin.

Postnatal brain OPCs attached to blood vessels that expressed the laminin isoforms. Thus, we next analyzed the attachment activity of OPCs to laminin α1, α2, α4, and α5 chains. We isolated Venus positive OPCs from P2-3 *Sox10*-Venus mouse brains and added them to wells coated with LME8s from laminin-111 (LM111E8), -211 (LM211E8), -411 (LM411E8), or -511 (LM511E8), and then counted attached cells after incubation for an hour. As shown in Fig. 2a, LM211E8, LM411E8, and LM511E8 exhibited OPC attachment activity. LM111E8 had no effect on adhesion of OPCs (Fig. 2a). When the cell attachment activity of LM411E8, one of the active LME8s, was compared with that of fibronectin, another extracellular matrix cell adhesion protein, the number of attached OPCs on LM411E8 was higher than that on fibronectin (Supplementary Fig. S1a). These results suggested that laminin α2, α4, and α5 chains promoted OPCs adhesion.

Laminins bind to the cell surface receptors including integrin α3β1, α6β1, α7β1, and α6β4^10^. The expression of integrin subunit α3, α6, α7, β1, and β4 in *Sox10*-Venus OPCs was confirmed by RT-PCR. Therefore, we tested the effect of functional blocking antibodies to integrin α3, α6, β1, and β4, except for α7, which was commercially unavailable, on OPC adhesion to LM211E8, LM411E8, and LM511E8 (Fig. 2b–d). We observed that OPC attachment on all of the three LME8s was significantly decreased in the presence of the blocking antibody to integrin β1 (Fig. 2b–d). These results indicated that OPCs adhesion to laminin α2, α4, and α5 chains was mediated via integrin β1.

### OPC migration activity of LME8s

**Figure 3:**
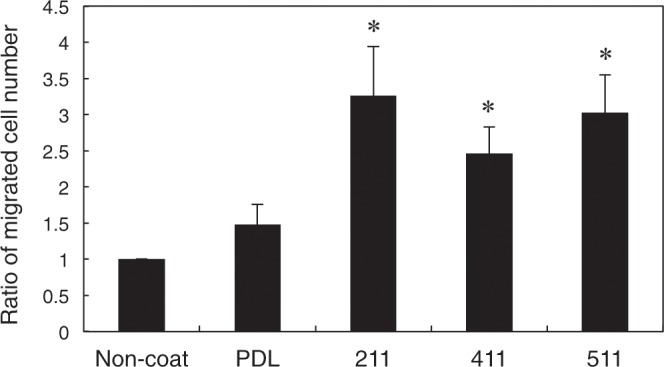
The effect of laminin E8 fragments on OPC migration. Ratio of migrated OPC numbers on recombinant laminin E8 fragments (LM211E8, LM411E8, and LM511E8) and poly-_D_-lysine. The number of migrated cells on non-coat as a control was set as 1.0. Error bars, s.e.m. (*p < 0.05, *t* test). At least triplicate experiments were independently performed. PDL: Poly-_D_-lysine; 211: LM211E8; 411: LM411E8; 511: LM511E8.

A recent study showed that OPCs migrate along blood vessels in the developing nervous system^11^. Also, one of the main functions of laminins is to promote migration of cells^4,5^. From the result that OPCs were associated with the brain vessels (Fig. 1), we hypothesized that the laminin isoforms regulate OPC migration. We therefore evaluated the effect of LM211E8, LM411E8, and LM511E8 on OPC migration using Transwell chambers (Fig. 3). As a consequence, the number of migrated OPCs on LM211E8, LM411E8, and LM511E8 was increased, compared with either a non-coated or poly-_D_-lysine (PDL)-coated chamber as a control, suggesting that OPC migration was promoted by laminin α2, α4, and α5 chains.

### OPC survival through FAK on LME8s

**Figure 4:**
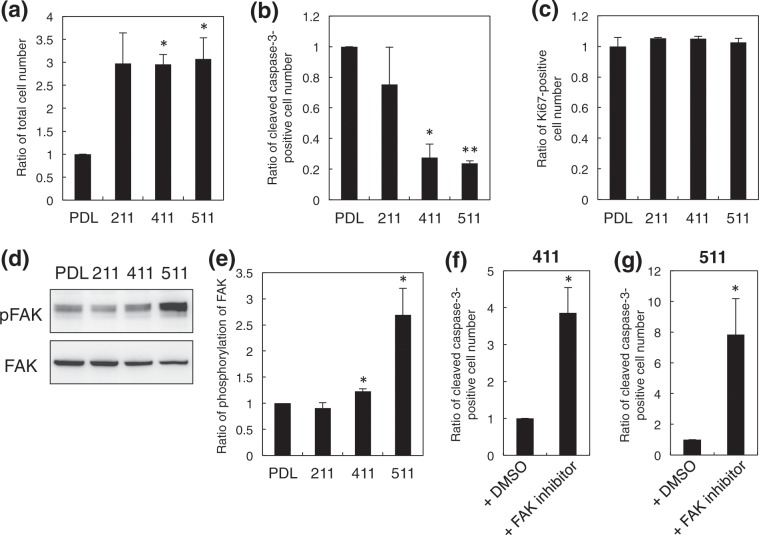
The effect of laminin E8 fragments on OPC survival. (**a**) Ratio of total OPCs numbers on recombinant laminin E8 fragments (LM211E8, LM411E8, and LM511E8). The number of attached cells on poly-_D_-lysine as a control was set as 1.0. Error bars, s.e.m. (*p < 0.05, *t* test). (**b**) Ratio of cleaved caspase-3-positive OPC numbers on recombinant laminin E8 fragments (LM211E8, LM411E8, and LM511E8). The number of cleaved caspase-3-positive cells on poly-_D_-lysine as a control was set as 1.0. Error bars, s.e.m. (*p < 0.05, **p < 0.01, *t* test). (**c**) Ratio of Ki67-positive OPC numbers on recombinant laminin E8 fragments (LM211E8, LM411E8, and LM511E8). The number of Ki67-positive cells on poly-_D_-lysine as a control was set as 1.0. Error bars, s.e.m. (**d**) Western blotting of phosphorylated FAK (Tyr397) and total FAK in OPCs cultured on recombinant laminin E8 fragments (LM211E8, LM411E8, and LM511E8). These images were cropped from the results of the membrane blots as indicated in Fig. S2. (**e**) Quantification of phosphorylation levels of FAK. The intensity of Western blotting bands was measured and pFAK/FAK was calculated. The phosphorylation level in OPCs on poly-_D_-lysine as a control was set as 1.0. Error bars, s.e.m. (*p < 0.05, *t* test). (**f,g**) Ratio of cleaved caspase-3-positive OPC numbers on LM411E8 (**f**) and LM511E8 (**g**) in the presence of the FAK inhibitor. The number of cleaved caspase-3-positive cells with only dimethyl sulfoxide as a control was set as 1.0. Error bars, s.e.m. (*p < 0.05, *t* test). At least triplicate experiments were independently performed. PDL: poly-_D_-lysine; 211: LM211E8; 411: LM411E8; 511: LM511E8; DMSO: dimethyl sulfoxide.

The interaction with laminins also promotes the proliferation and survival of cells^4,5^. Thus, we cultured OPCs for 3 days on LM211E8, LM411E8, and LM511E8 and counted live cell numbers. After the 3-day-culture, the cell numbers were decreased in all the conditions, compared with those on the 1st day of the culture. We found that the cell number on LM411E8 and LM511E8 after the culture for 3 days was more than the control PDL, whereas LM211E8 also showed more cell number without a statistical difference (Fig. 4a). We next analyzed the expression of cleaved caspase-3 and Ki67 in OPCs by immunostaining to label apoptotic and proliferative cells, respectively (Fig. 4b,c). The immunostaining showed that the percentage of cleaved caspase-3-positive OPCs was significantly decreased on LM411E8 and LM511E8, but not on LM211E8, in comparison with PDL, although there was no difference in immunostaining of Ki67 (Fig. 4b,c). In addition, the number of apoptotic OPCs on LM411E8 was smaller than that on fibronectin (Supplementary Fig. S1b). From these results, laminin α4 and α5 chains promoted survival of OPCs.

Downstream of integrins, several signaling molecules, including FAK, are activated to promote adhesion-dependent cell survival^12^. When the phosphorylation of Tyr397 in FAK, which is the most critical tyrosine residue in FAK activation, was examined in OPCs cultured on LME8s, LM511E8 elevated the phosphorylation level of FAK (Figs. 4d,e and S2). Also, the slight but significant increase of FAK phosphorylation was observed on LM411E8 (Figs. 4d,e and S2). Further, we tested an inhibitor of FAK in the OPC culture on LM411E8 and LM511E8. The FAK inhibitor substantially increased the number of dying apoptotic cells labeled by cleaved caspase-3 (Fig. 4f,g). These experimental data indicated that laminin α4 and α5 chains activated FAK for survival of OPCs. We finally examined a difference of the activities between LM411E8 and LM511E8, since the phosphorylation level of FAK in OPCs on LM511E8 was higher than that on LM411E8 although there was no difference in cell survival. The phosphorylation levels of Akt, a critical downstream molecule of FAK for cell survival, were similar between LM411E8 and LM511E8 (Supplementary Fig. S3). However, we found that the cell process formation with filopodia-like protrusions was higher on LM511E8 than on LM411E8, accompanying with an increase of filamentous actin-positive area (Supplementary Fig. S4). These results suggested that laminin α5 chain activated the signaling of actin polymerization, probably through the activation of FAK, for cell process formation.

## Discussion

In this study, we showed that the expression of laminin α1, α2, α4, and α5 chains was observed surrounding blood vessels at the early postnatal brain and OPCs attached to the vessels expressed these laminin isoforms. The expression of laminin α chains in basement membranes around vessels is known in other tissues. The blood vessels are distinguished into four types with its distinct structures: arterioles, capillaries, postcapillary venules, and venules^13^. Arterioles and venules are surrounded by endothelial and perivascular basement membranes located beneath endothelial cells and peripherally smooth muscle cells, respectively. Capillaries and postcapillary venules are underlain only by endothelial basement membranes that embed pericytes. Laminin α4 chain is expressed in all of the basement membranes, while the expression of α2 chain is observed in only perivascular basement membranes^14,15^. Laminin α5 chain is found in most of the basement membranes but its expression is patchy in endothelial basement membranes around postcapillary venules and venules^16^. Here we found that laminin α4 chain was expressed in all of CD31-positive vessels in the postnatal brain, while about 15% of the vessels were positive for α2 and α5 chains, indicating that laminin α2, α4, and α5 chains may be expressed with similar pattern to that in the other tissues. We also detected the expression of laminin α1 chain in some of the vessels, whereas LM111E8 did not show attachment activity of OPCs. Laminin α1 chain may possess different functions from the other chains. In the previous report, laminin β1 and γ1 were found in the VZ/SVZ, in addition to vascular basement membranes, at P1, while the expression of laminin α chains was not investigated^6^. In the VZ/SVZ of the adult mouse brain, laminins are detected in basement membranes of fractones, which are the extravascular protrusion-like structure, and provide a niche for neural stem cells^17^. During the postnatal stages, the protein expression of laminin α5 chain was detected in the SVZ fractones at P3, but not at P0^18^. In the present study, we could not detect the clear expression of laminin α chains in the VZ/SVZ at P2. However, there is a possibility that the initiated expression of laminin α chains in fractones may influence on OPC activity.

Laminins exert biological activities, such as cell migration and survival, through cell adhesion mediated by integrins^4^. Our *in vitro* experiment of the cell attachment assay revealed that LME8s containing laminin α2, α4, and α5 chains promoted OPCs adhesion and anti-integrin β1 antibody inhibited the adhesion. From these data, integrin β1 was significantly involved in the attachment to the laminins on OPCs. In the present study, integrin α chain as a partner of integrin β1 to recognize the laminins on OPCs was unable to be determined. It is possible that a combination of more than two different α chains tested here formed heterodimers with integrin β1 and function as receptors for these laminins, or OPC adhesion to the laminins was mediated via the other integrin α chain, which we did not examine in this study.

LM211E8, LM411E8, and LM511E8 promoted OPC migration, and LM411E8 and LM511E8 maintained the number of OPCs through the suppression of apoptosis, but not promotion of cell proliferation. During the embryonic stages, OPCs migrate to their destination along on blood vessels as rails^11^. Laminin α2, α4, and α5 chains may have an activity for guiding OPCs to the location where they need to settle. OPC survival is prolonged by growth factors, such as platelet-derived growth factor (PDGF) and fibroblast growth factor-2 (FGF2)^19,20^, which were contained in the medium used in this study. Binding of laminins to cell surface integrins induces the formation of the molecular platform, such as focal contact including FAK, for intracellular signaling^12^. The growth factor receptors are recruited to the platform, and efficiently bind to the growth factors and activate downstream pathways for cell survival^21^. Our data showed that the FAK inhibitor increased apoptotic OPCs on LM411E8 and LM511E8. These evidences suggest that laminin α4 and α5 chains enhance OPC survival, presumably on blood vessels, with positively regulating the downstream FAK signaling. Furthermore, the more elevated activation of FAK in OPCs on LM511E8 than on LM411E8 likely activated cell process formation with actin polymerization. OPCs probably choose the signaling from these different laminin α chains, depending on their requirements.

*In vivo* functional study of laminin α2 chain in the postnatal OPC development has been previously carried out. OPC survival, but not proliferation, is reduced in the VZ/SVZ of laminin α2 null mice during the early postnatal stage^6^. In the present study, the number of OPCs on LM211E8 was more than that on the control PDL after the 3 day-culture, though without a statistical difference. Also, no significant difference in the ratio of cleaved caspase-3-positive OPC numbers was observed between LM211E8 and PDL. These observations suggest that laminin α2 chain promotes OPC survival, but some *in vivo* factors are required for exerting the activity. Knockout mice of laminin α4 chain exhibit defects in Schwann cell development and nerve fasciculation in the peripheral nervous system, in addition to the abnormalities in neuromuscular junction formation^22,23^. However, no defects were found in the laminin α4 deficient CNS^22,23^. To explain this, laminin α5 chain probably compensates for the deficiency of laminin α4 chain, since an ectopic expression of laminin α5 chain is observed around all vessels instead of laminin α4 chain^24^. Therefore, double knockout of laminin α4 and α5 chains in the CNS tissue would be required for analyzing the postnatal development of OPCs, whereas it is technically difficult due to the embryonic lethality of laminin α5 knockout mice and the expression of laminin α5 chain in multiple cell types in the CNS^25,26^.

During the differentiation from OPCs to oligodendrocytes, the signaling of laminin α2 chain, which is expressed on axon surface in the brain stem and cervical spinal cord, has been characterized^27,28^. Differentiating oligodendrocytes adhere to the axonal surface via the binding of integrin α6β1 to laminin α2 chain (laminin-211), which results in the switching of intracellular signaling from the phosphoinositide 3-kinase/Akt pathway for proliferation to the mitogen-activated protein kinase-dependent pathway for survival, in the presence of soluble neuregulin-1^27^. From these observations and our data in the present study, laminin α2, α4, and α5 chains surrounding vessels regulate migration and survival of OPCs, but later, laminin α2 chain on axonal surface promotes oligodendrocyte survival during the differentiation and myelination stage, particularly together with neuregulin-1, which is expressed and released from axons.

Fibronectin is an extracellular matrix protein that promotes cell adhesion as well as laminins. However, fibronectin does not promote the survival of differentiating oligodendrocytes, whereas laminin-211 does as mentioned above^27,29^. Previously, Hu *et al*. revealed that the proliferation, survival, and migration of OPCs were increased on fibronectin, similar to laminin^30^. In their study, however, laminin-111, whose E8 (LM111E8) did not exhibit attachment activity of OPCs in our present study, was used^30^. Also, our data showed that fibronectin was less active than LM411E8 in both cell attachment and survival assays. From these, laminins, but not fibronectin, are a positive regulator in the survival of either OPCs or oligodendrocytes. It is interesting to note that fibronectin as one of components of extracellular vesicles, which are secreted from microvascular endothelial cells, promotes the uptake of the vesicles into OPCs for their proliferation and survival^31^. Fibronectin may exert its activity as a component of the vesicles, but not as a matrix protein. In addition, rather than promoting effect, fibronectin negatively regulates process outgrowth in differentiating oligodendrocytes, which is associated with an attenuation of matrix metalloproteinase-9 activity^32,33^. Lafrenaye and Fuss discussed that the coordination of these opposed functions between laminin-211 and fibronectin was required for progression of the oligodendrocyte differentiation with proper timing^33^. The balances of the coordinated activities of these extracellular proteins probably determine OPC and oligodendrocyte behaviors and their fate.

In this study, we used LME8s, instead of intact laminins. LME8s possess the integrin binding activity with the same level as intact laminins, and can be prepared more easily and efficiently than intact laminins. Under the pathological condition such as demyelination, the replenishment of OPCs with their migration and survival is required for remyelination. Previous studies revealed that OPCs population was depleted after acute demyelination^34^ and are not able to catch up the fast demand of OPCs under chronic conditions^35^. LME8s may be useful reagents to facilitate an improvement of the pathological condition. In conclusion, our study revealed biological functions of laminin α chains on survival and migration of OPCs. This is the first investigation that evaluated all the laminin α chains in OPC development and revealed their activities. The results in this study will give better understanding of OPC biology and development of OPC culture methods and of therapeutic reagents for OPC related disorders.

## Methods

### Animals

P2-3 pups from the previously reported *Sox10*-Venus mice^9^ and P2 WT mice were euthanized by decapitation as described in the International Animal Care and Use Committee and the previous report^36^, and were used for the experiments. All procedures for animal experiments were approved by the Tokyo Medical and Dental University Animal Care and Use Committee (Protocol No: A2019181) and the Juntendo University School of Medicine Animal Care and Use Committee (Institutional review board No. 270050). All methods were conducted in strict accordance with the approved guidelines of the institutional animal care committees.

### Immunohistochemistry

P2 WT and P2 *Sox10*-Venus mice were anesthetized and perfused transcardially with phosphate buffered saline (PBS) and then with 4% paraformaldehyde (PFA; FUJIFILM Wako Pure Chemical) in PBS. The brains were collected and post-fixed with 4% PFA over night at 4 °C and then cryoprotected for 48 hours in gradient sucrose (15% and 30%) at 4 °C. The brains were embedded in OCT compound (Sakura Finetek), and frozen in dry ice. The embedded brain tissues were sectioned at 6 μm using a cryostat (Leica). The frozen sections were air dried and fixed for 10 minutes in 100% methanol at −20 °C or in 4% PFA at room temperature. The sections fixed with 4% PFA were carried out target retrieval with Target Retrieval Solution (Agilent). After blocking with blocking buffer (10% goat serum in PBS) for 1 hour at room temperature, they were incubated with primary antibodies overnight at 4 °C. The slides were washed with PBS and bound primary antibodies were labeled with fluorescence-conjugated secondary antibodies for 45 minutes at room temperature. After several washes, sections were mounted in mounting medium for fluorescence with DAPI (Vector Laboratories). Primary and secondary antibodies were as follows: rat anti-laminin α1, laminin α3, laminin α4, laminin α5^7^, rat anti-laminin α2 (Sigma-Aldrich), rabbit anti-CD31 (Abcam), rabbit anti-Olig2 (Merck Millipore), rat IgG-Alexa488 (ThermoFisher Scientific), rat IgG-Alexa555 (ThermoFisher Scientific), and rabbit IgG-Alexa594 (ThermoFisher Scientific).

### Primary cell preparation

Primary mixed cell cultures were prepared from the brain of P2-3 *Sox10*-Venus mice. The whole brain was dissected out and the meninges were removed. Then, the brains were diced into 1 mm^3^ chunks in a dish with ice-cold Leibovitz’s L-15 medium (ThermoFisher Scientific) and enzymatically dissociated with 10 ml of papain/DNase I solution containing: 40 mg of α-_D_-Glucose (Sigma-Aldrich), 4 mg of bovine serum albumin (BSA; Sigma-Aldrich), 4 mg of _L_-cysteine (Sigma-Aldrich), 2.5 μl/ml papain (Worthington), and 5 μl/ml DNase I (Sigma-Aldrich) in PBS. After an incubation for 20 minutes at 37 °C, the cells were collected by centrifugation in a swinging bucket at 100 × *g* for 5 minutes. The supernatant was removed and Dulbecco’s modified Eagle’s medium (DMEM; ThermoFisher Scientific), supplemented with 10% fetal bovine serum (FBS; ThermoFisher Scientific), as well as sodium pyruvate (Sigma-Aldrich), _L_-glutamine (ThermoFisher Scientific), and 100 units/ml penicillin and 100 μg/ml streptomycin (ThermoFisher Scientific) were added. The pellet was dissociated, and the tissue suspension was filtered through a 70 μm nylon cell strainer (BD Biosciences).

### Isolation of OPCs using flow cytometry

The cell suspension was collected in a 5 ml tube with cell-strainer cap (BD Biosciences) and was labeled with propidium iodide (Sigma-Aldrich) to remove cell debris and dead cells. Cell sorting was performed using a MoFlo flow cytometer (Beckman Coulter) and isolated *Sox10*-Venus positive cells as OPCs as described previously^9^. Sorted OPCs were used for each experiment.

### Cell attachment and inhibitory assay

Immulon 2HB 96-well plate (ThermoFisher Scientific) was coated with LME8 (LM111E8, LM211E8, LM411E8, or LM511E8) at 30 nM. The substrate-coated plates were incubated at 4 °C overnight. After blocking with 1% BSA for 1 hour at 37 °C, sorted Venus positive OPCs in 0.1% BSA/DMEM were plated on coated wells at 1.0–2.0 × 10^4^ cells per well and incubated for 1 hour at 37 °C. The attached cells were stained with 0.2% crystal violet aqueous solution in 20% methanol for 10 minutes at room temperature and counted. In the inhibitory assay, the cell suspension was pre-incubated at 37 °C for 15 minutes with 10 μg/ml of the inhibitory antibody. Antibodies were as follows: anti-α3 integrin (clone name: P1B5; Merck Millipore), anti-α6 integrin (clone name: NKI-GoH3; Merck Millipore), anti-β1 integrin (clone name: 9EG7; BD Biosciences), anti-β4 integrin (clone name: ASC-9; Merck Millipore) and normal rat IgG (Santa Crus Biotechnology) as a control.

### Migration assay of OPCs

Migration of OPCs was performed using a 24-well Transwell chamber with 8 μm pore-size (Corning). Lower filters were coated with LM211E8, LM411E8, or LM511E8 at 1 μg/ml, and control PDL (Sigma-Aldrich) at 10 μg/ml. Isolated OPCs in OPC Medium [Basal chemically defined medium with 10 ng/ml PDGF-AA (PeproTech), 10 ng/ml FGF2 (PeproTech) and 100 units/ml penicillin and 100 μg/ml streptomycin^36^] with 0.075 μg LME8s were plated on the upper wells at 1.0–2.0 × 10^4^ cells per well. After 24 hours of culture at 37 °C, cells on the upper surface of the membrane were removed, whereas migrated cells on the lower membrane surface were fixed in 100% methanol for 15 minutes and stained in 0.2% crystal violet aqueous solution in 20% methanol for 20 minutes and counted.

### Culture of OPCs

Isolated OPCs were plated in 8-well chamber slides (Matsunami Glass) or 12-well culture slides (Matsunami Glass) coated with LM211E8, LM411E8, LM511E8, or PDL as a control at 1.5 × 10^4^ cells per well and incubated at 37 °C for 3 days. OPCs were cultured in OPC Medium. In the inhibition experiment, 10 μM Focal Adhesion Kinase Inhibitor II (Merck Millipore) dissolved in DMSO was used.

### Immunocytochemistry

Cultured cells were fixed in 4% PFA for 10 minutes at room temperature and permeabilized with 0.1% Triton X-100 (Sigma-Aldrich) in PBS for 10 minutes at room temperature for detection of intracellular proteins. After blocking with Power Block Universal Blocking Reagent (BioGenex Laboratories) for 1 hour at room temperature, they were incubated with primary antibodies overnight at 4 °C. The slides were washed with PBS and incubated with secondary antibodies for 45 minutes at room temperature and mounted. Primary and secondary antibodies were as follows: rabbit anti-cleaved caspase-3 (Cell Signaling), rabbit anti-Ki67 (Leica), and rabbit IgG-Alexa 594.

### Analysis of cell morphology and cell count

For antigenic phenotyping, positive cells for each antigen were counted and expressed as a percentage out of all Venus positive cells. Confocal microscopy images were obtained using a Leica TCS-SP5 confocal laser scanning microscope (Leica), and all confocal settings were set to the same parameters for each experiment. Metamorph software was used for analysis, and at least three independent sorting experiments were analyzed. The numbers were expressed as means ± standard error.

### Western blotting

The cell lysates from OPCs on LME8s were prepared and the protein samples were analyzed by Western blotting as described previously^37^. Antibodies against phospho-FAK (Tyr397) (ThermoFisher Scientific) and FAK (Merck Millipore) were used. Measuring intensity of Western blotting bands was performed using Image J.

## Supplementary information

Supplementary Information

## Data Availability

All the data are presented in the manuscript as figures.
