# Supplementary material for: Laminin α2, α4, and α5 Chains Positively Regulate Migration and Survival of Oligodendrocyte Precursor Cells
Source: Sci Rep. 2019 Dec 27;9:19882. doi: 10.1038/s41598-019-56488-7 (PMC6934537; doi:10.1038/s41598-019-56488-7)
Supplement: Supplementary file 1 — Supplementary Information [file 41598_2019_56488_MOESM1_ESM.pdf]

Supplementary Information

Laminin α2, α4, and α5 Chains Positively Regulate Migration and Survival of Oligodendrocyte Precursor Cells

Nobuharu Suzuki[1,2†*], Mai Hyodo[2†], Chikako Hayashi[1,2†], Yo Mabuchi[2,3], Kaori Sekimoto[2], Chinami Onchi[1], Kiyotoshi Sekiguchi[4], and Chihiro Akazawa[2,3*]

[1]Department of Molecular and Cellular Biology, Graduate School of Medical and Dental Sciences, Tokyo Medical and Dental University (TMDU), Tokyo, Japan
[2]Department of Biochemistry and Biophysics, Graduate School of Health Care Sciences, TMDU, Tokyo, Japan
[3]Department of Biochemistry and Biophysics, Graduate School of Medical and Dental Sciences, TMDU, Tokyo, Japan
[4]Division of Matrixome Research and Application, Institute for Protein Research, Osaka University, Suita, Osaka, Japan

*To whom correspondence should be addressed: Nobuharu Suzuki, Ph.D., Department of Molecular and Cellular Biology, Graduate School of Medical and Dental Sciences, TMDU, 1-5-45 Yushima, Bldg 3, Bunkyo-ku, Tokyo, Japan, 113-8510, E-mail: nsuzbb@tmd.ac.jp, Pnone: +81-3-5803-5364; Fax: +81-3-5803-5364, and Chihiro Akazawa, MD, Ph.D., Department of Biochemistry and Biophysics, Graduate School of Medical and Dental Sciences, TMDU, 1-5-45 Yushima, Bldg 3, Bunkyo-ku, Tokyo, Japan, 113-8510, E-mail: c.akazawa.bb@tmd.ac.jp, Pnone: +81-3-5803-5362; Fax: +81-3-5803-5362

†These authors equally contributed to this work.

Supplementary Figure S1: Cell attachment and survival activity of OPCs on fibronectin and LM411E8.

Supplementary Figure S2: Images of whole membranes of Western blotting indicated in Fig. 4d.

Supplementary Figure S3: Phosphorylation of Akt on LM411E8 and LM511E8.

Supplementary Figure S4: Cell morphology and actin organization in OPCs on LM411E8 and LM511E8.

Figure S1

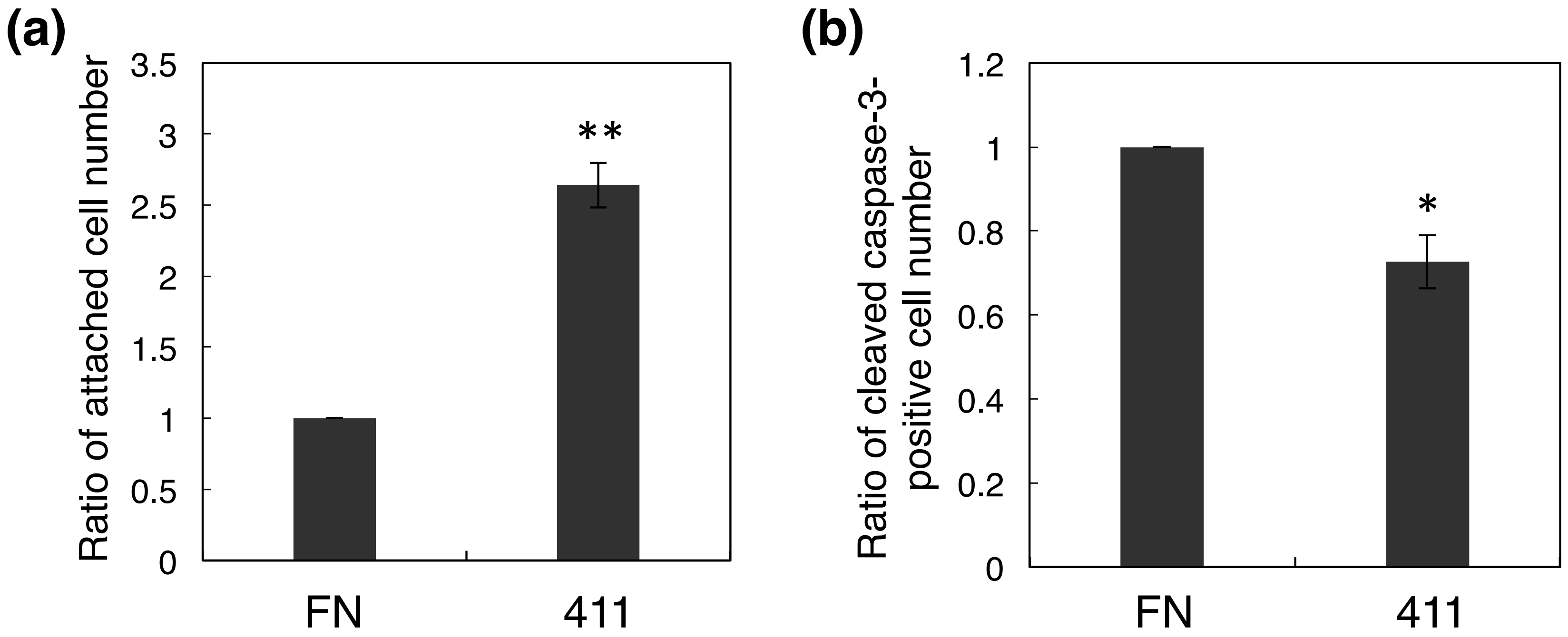

Supplementary Figure S1. Cell attachment and survival activity of OPCs on fibronectin and LM411E8. (a) Ratio of attached cell numbers on fibronectin and LM411E8 (The concentration of the proteins was 30 nM). The number of attached cells on fibronectin was set as 1.0. Error bars, s.e.m. (**$p < 0.01$, *t* test). (b) Ratio of cleaved caspase-3-positive OPCs numbers. The number of cleaved caspase-3-positive cells on fibronectin was set as 1.0. Error bars, s.e.m. (*$p < 0.05$, *t* test). Triplicate experiments were independently performed. FN: fibronectin; 411: LM411E8.

Figure S2

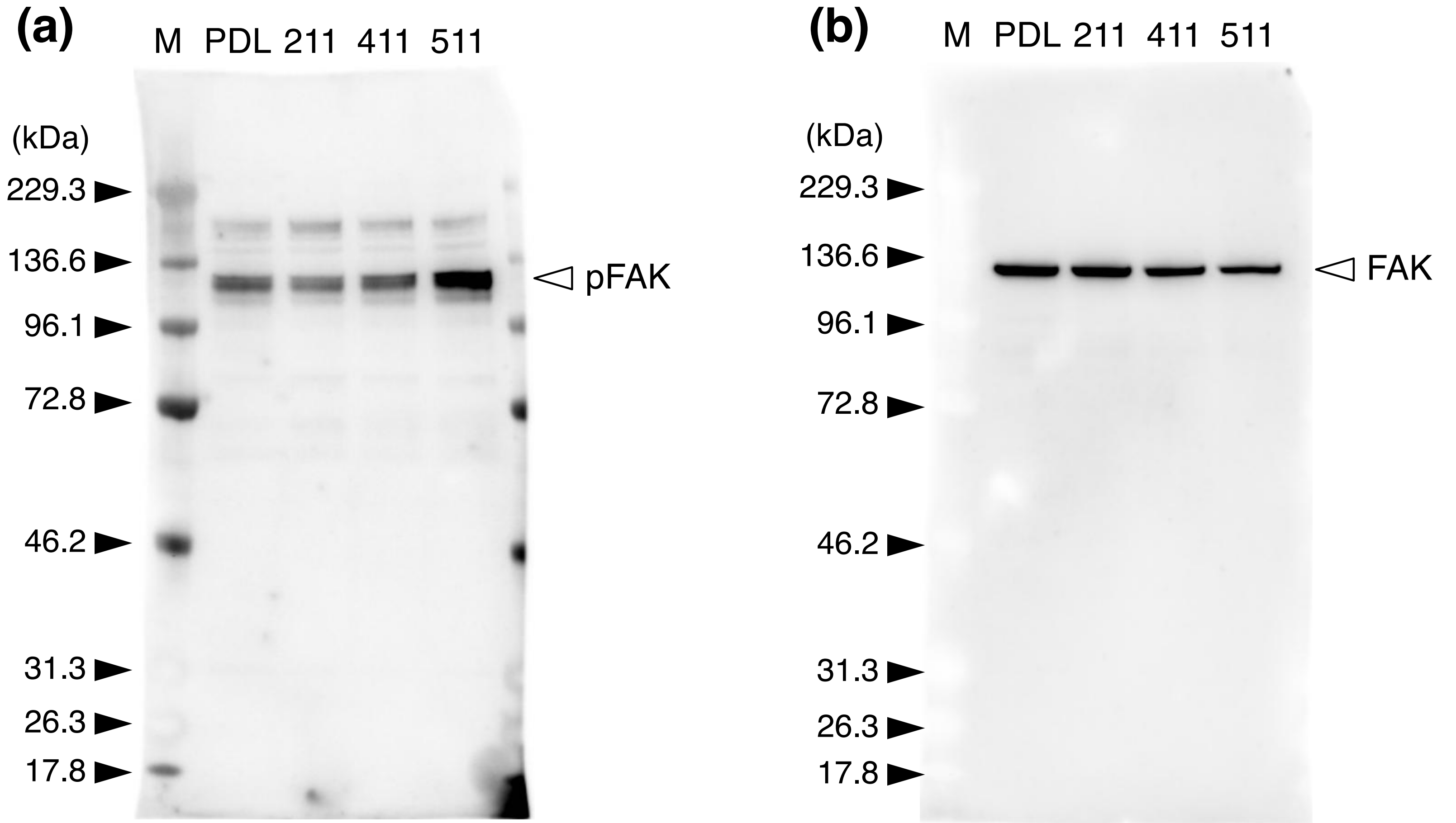

Supplementary Figure S2. Images of whole membranes of Western blotting indicated in Fig. 4d. Western blotting of pFAK (a) and FAK (b) using the protein samples of OPCs cultured on LME8s was carried out as described in Methods. Western blotting images were taken using Amersham Imager 600 with the following exposure time: 1 minute for pFAK (a) and 2 minutes and 53 seconds for FAK (b). The expected molecular weight of FAK is 125 kDa. M: Protein size marker (DM637, BioDynamics Laboratory); PDL: Poly-$_{D}$-lysine; 211: LM211E8; 411: LM411E8; 511: LM511E8.

Figure S3

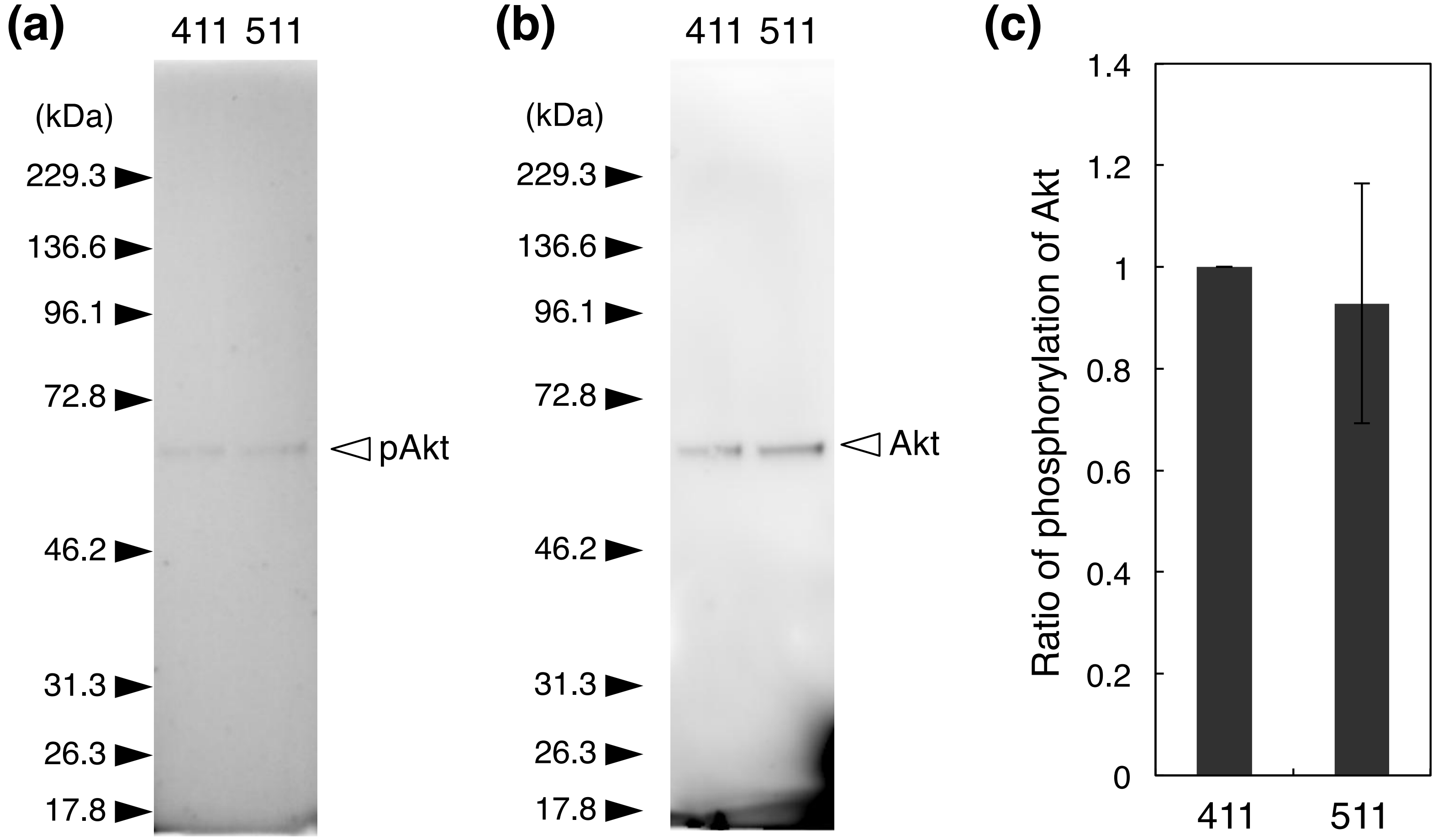

Supplementary Figure S3. Phosphorylation of Akt on LM411E8 and LM511E8. Western blotting of pAkt (a) and Akt (b) using the protein samples of OPCs cultured on LM411E8 and LM511E8 was carried out as described in Methods. Western blotting images were taken using Amersham Imager 600 with the following exposure time: 11 minute for pAkt (a) and 15 minutes for Akt (b). The expected molecular weight of Akt is 60 kDa. M: Protein size marker (DM637, BioDynamics Laboratory). (c) Ratio of phosphorylation levels of Akt. The intensity of Western blotting bands was measured and pAkt/Akt was calculated. The phosphorylation level in OPCs on LM411E8 was set as 1.0. Error bars, s.e.m. Triplicate experiments were independently performed. 411: LM411E8; 511: LM511E8.

Figure S4

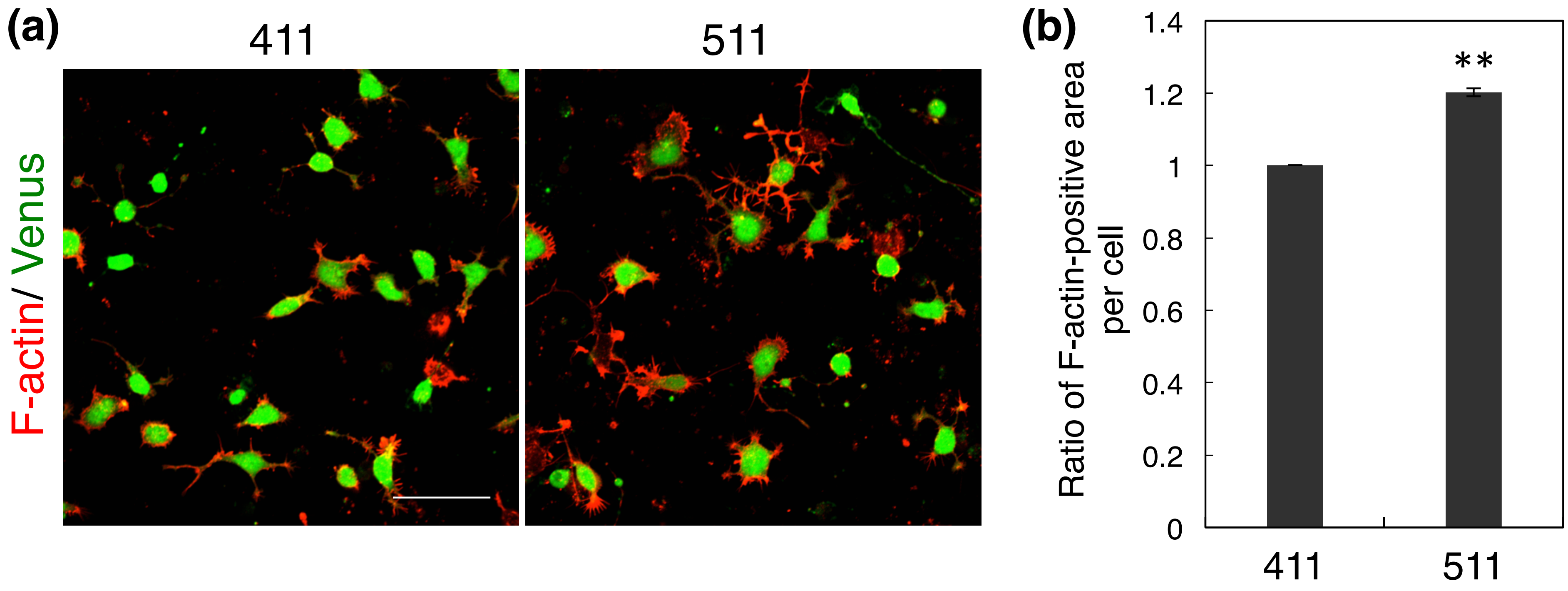

Supplementary Figure S4. Cell morphology and actin organization in OPCs on LM411E8 and LM511E8. (a) Immunocytochemistry of filamentous actin (red) in *Sox10*-Venus mouse OPCs on LM411E8 and LM511E8 (The concentration of the proteins was 30 nM). Scale bars: 50 µm. (b) Ratio of F-actin-positive area per OPC. The F-actin-positive area on LM411E8 was set as 1.0. Error bars, s.e.m. (**p < 0.01, t test). Triplicate experiments were independently performed. 411: LM411E8; 511: LM511E8; F-actin: filamentous actin.
